# High spin chemistry underlying organic molecular magnetism: Topological symmetry rule as the first principle of spin alignment in organic open-shell systems of *π*-conjugation and their ions

**Published:** 2004-02-01

**Authors:** Koichi Itoh, Takeji Takui

**Affiliations:** Departments of Materials Science and Chemistry, Graduate School of Science, Osaka City University, Sugimoto, Sumiyoshi-ku, Osaka 558-8585

**Keywords:** High spin chemistry, ***π***-topological symmetry rule, organic high-spin molecule, molecule-based magnetism, organic ferromagnets, electron magnetic resonance

## Abstract

This review paper deals with an overview of molecule-based magnetism as a rapidly developing interdisciplinary field, topological symmetry rule as the first principle of spin alignment in organic open-shell systems in the ground state, the proposal of organic through-bond 1D and 2D ferro- and superparamagnets and the detection of the first organic high-spin molecule, *m*-phenylenebis(phenylmethylene) in the quintet ground state (*S* = 2), followed by extended organic high-spin systems with ***π***-conjugation such as aromatic hydrocarbons having *S* = 3, 4, 5. The paper also describes a theoretical approach to the understanding of electronic spin structures of organic high-spin molecules by invoking both Heisenberg and Hubbard model Hamiltonians, weakly interacting intramolecular high-spin systems from both experimental and theoretical sides, the spin density distribution of the first organic high-spin molecule in terms of electron- nuclear multiple resonance spectroscopy and the detection and characterization of ionic high-spin hydrocarbons, emphasizing the establishment of high spin chemistry underlying organic molecular magnetism.

## Introduction

High spin chemistry underlying organic molecule-based magnetism dates back to the detection of the first organic high-spin molecule in its ground state in 1967.1) The field of organic molecule-based magnetism and materials science (abbreviated to organic magnetics)2)–7) has attracted widely increasing interest from both the pure and applied natural sciences for the last decades.4)–7) This is not only due to the rich variety of novel physical phenomena and properties which synthetic organomagnetic materials are expected to exhibit both macro- and meso-scopically, but also due to their underlying potential applications as future spin science and spin technology in chemistry and materials science, such as spin-mediated molecular electronics, quantum computer devices, molecular spinics7) and so on.8) Organic magnetics has implied that the magnetics is characteristic of “system-properties” and extends for research areas studying spin assemblages and related novel quantal phenomena appearing on a semi-macroscopic scale (molecular spinics). Molecular spinics is spin manipulation science and electronic spin technology based on spin-polarized nature and quantal waves.7) The spinics underlies tera-chemistry in which molecular ensembles and their cooperative wave nature appearing in a semi-macroscopic scale are treated. Organic magnetics is such a frontier encompassing a cross-disciplinary field of future science and technology. New generation of organic magnetics has emerged since 1995 and over the past several years multi-functionality magnetic materials of hyperstructured molecules or novel quantal magnetic phenomena originating in spin assemblages in a micro- and semi-macroscopic scale have been searched for in various levels of research.

A rapid development of this research field is due to the fact that the study of organic magnetics has given rise to important conceptual advances in chemistry and physics. Purely organic magnetics originating in purely organic building spin systems composed only of light atoms such as C, H, N, O, and S, is apparently a controversial issue simply because organic substances are intrinsically diamagnetic. The search for organic magnetic materials, however, is not only the focus of the contemporary topics in materials science, but also an important issue of the interplay between theory and experiment in physics and rapidly developing spin chemistry.2)–7) It is not until as early as 1960’s when a topological through-bond approach to organic ferromagnetics 2) that the possible occurrence of organic ferromagnetics emerged and high spin chemistry was disclosed which is now a dominant branch of molecular spin science.9)–10) In view of history of magnetics, the topological approach has been the most controversial issue against a famous common scientific statement by Heisenberg that ferromagnetics is intrinsic to inorganic materials composed of d-electron assemblages and therefore 2s- and 2p-electrons have nothing to do with ferromagnetics.

## Essential difference between conventional atom-based magnetics and molecule-based magnetics

One of the easy ways to understand what organic magnetics is and to characterize the present stage of the research is to compare molecule-based magnetics with conventional atom-based magnetics in terms of important indices.7) In this section, essential difference between molecule-based magnetics and conventional atom-based magnetics is briefly described.

**Fig. 1:**
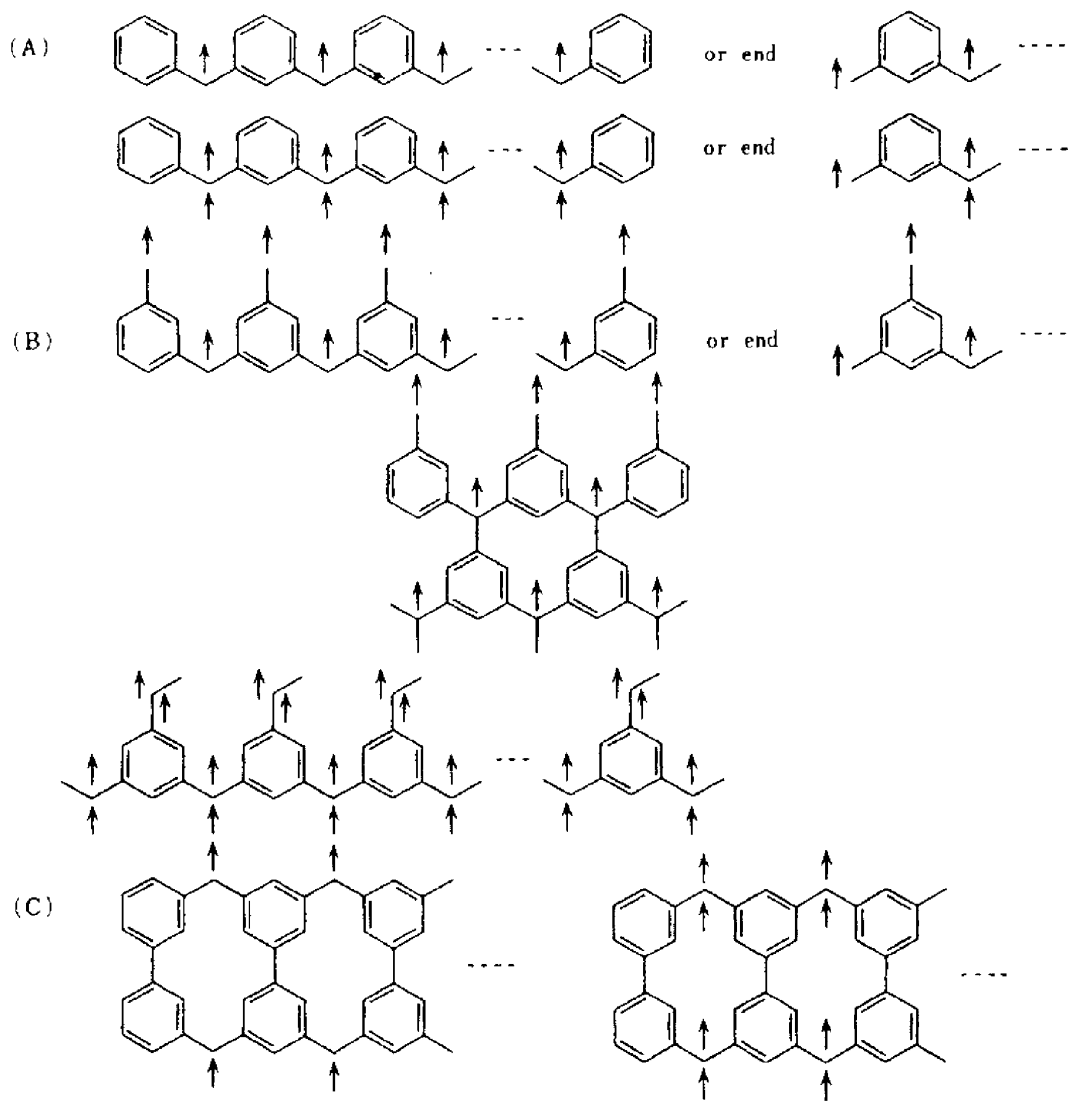
Through-bond superparamagnetic and ferromagnetic hydrocarbon-based polymers proposed at early times in terms of the robust spin polarization.2a)–2c) It was shown later on that the polymers (C) were not the case.2d),2f)

**Fig. 2:**
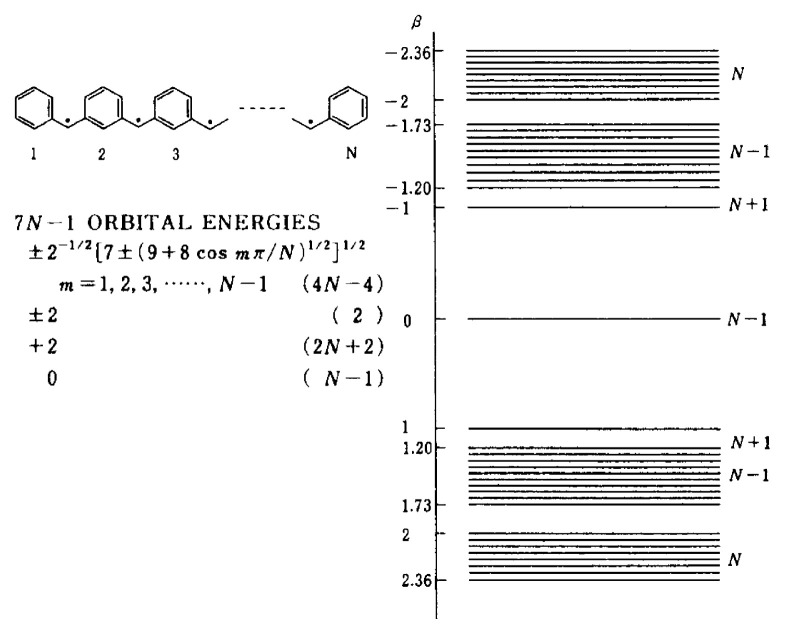
The first documented band-structure calculation for an *m-*benzyl-based polymeric spin system.2a),2c) An analytical solution in terms of Hueckel Hamiltonian is given. The NBMO band at zero energy is superdegenerate.

Molecular design is essential in organic magnetics in which spin bearing units are molecules composed of s and p electrons instead of transition metal ionic basis of one center of d and f electrons. This arises from the fact that any organic molecular framework is multi-centered. Because of this reason the intrinsic conceptual ground of ***π***-topological symmetry requirement is underlain on the theoretical side.2) Group theoretical arguments never give the unlimitedness of molecular orbital degeneracy (see Figs. 1 and 2), which is led by topological symmetry arguments of ***π***-electron networks (connectivity symmetry of ***π***-conjugation). The exploitation of the topological nature has contrasted organic magnetics with conventional atom-based magnetics. Requirements of molecular design in organic magnetics are closely related to the multi-centered nature of molecules as spin carriers. In this context, Heisenberg-Dirac Hamiltonian approaches11) are only phenomenological and for some cases microscopic details as molecules have to be restored for physical interpretations of observed static as well as dynamic magnetic properties.

## Genealogical survey of molecular design for organic magnetics; through-bond vs. through-space approach

The first conceptual advance in organic ferromagnetics can date back to the two proposals of its possible occurrence on the theoretical side.2),3) The two proposals are based upon completely different theoretical grounds. The molecular design of the first one exploits a through-bond approach which is based on the topological nature of ***π***-conjugation networks in aromatic hydrocarbons2) (through-bond approach/Itoh-Mataga’s approach). In the history of magnetics, the through-bond approach for the first time emerged to give a testing ground for establishing organic ferromagnetics.12)–14) The other proposal exploits intermolecular interaction such as charge-transfer interaction between organic donors and acceptors, where subtle crystal packing modes are required to be controlled and tuned in crystalline solids3) (through-space approach/McConnell-Breslow’s approach).15) It is not until recently that the first purely organic crystalline ferromagnet (Curie temperature *T*c ≅ 0.6 K) based on a through-space approach has been found (Kinoshita-Sugano-Awaga model).4),16) The molecular building spin block was a neutral doublet radical, *p*-nitrophenyl nitronyl nitroxide.16) The through-space approach associated with crystal packing modes includes Yamaguchi model where CT components have stable doublet radical moieties.12a)–12d) Yamaguchi model can be termed as a CT-radical hybrid approach while the through-space approach originally proposed by McConnell3a) and various modified varieties17)–20) can be termed as genuine CT approaches.

Recently, a novel through-bond approach to polymeric organic ferromagnets has been proposed21) (polaronic ferromagnet approach), where doping processes of diamagnetic polymers designed in terms of the ***π***-topological symmetry of conjugated systems give rise to ferromagnetically coupled spins induced in the ***π***-systems. Molecular design of diamagnetic polymers employed in this approach is also based upon the ***π***-topological symmetry argument which is essentially the same as in the topological spin polarization approach (Itoh-Mataga’s approach). One of the strong motivations behind Fukutome’s polaronic ferromagnet approach is to find a way to organic ferromagnetism without exploitation of chemically reactive spin sites during the course of synthetic or preparation processes. A challenging and elaborate experiment by Dougherty’s group has demonstrated the occurrence of high-spin ground states from a doped polymer.22) It turned out that the magnetic polymer employed was air-sensitive, hampering the originally proposed advantage. This through-bond approach is required to undergo further detailed evaluation of magnetic properties and electronic spin structures of model polymers from the microscopic viewpoint.

## Through-bond approach based on topologically controlled *π*-spin polarization

### The first documented through-bond approach to organic ferroand superpara-magnetics

Naturally, organic magnetics appearing in various forms of macroscopic scale involves cooperative phenomena and phase transitions which are inherently related to infinite or macroscopically extended length of coherence in magnetic interactions of organic materials. In view of polymeric organic magnetic materials, it should be emphasized that the first conceptual advance in the research field of organic magnetism was made in the context of a proposal of hypothetical one- or two-dimensional organic polymer large spins (assemblages of a great number of exchange-coupled spins), as shown in Fig. 1.2a)–2c)

The first documented through-bond approach to organic ferromagnetics appeared at early times exploited the topological nature of the symmetry of ***π***-conjugated electron network in organic molecules, which renders the degeneracy of singly occupied ***π***-nonbonding MO’s unlimited (the topological degeneracy of the nonbonding MO’s associated with topological robust ***π***-spin polarization as an underlying mechanism of organic ferromagnetic states).2a)–2c) In contrast, the degree of the orbital degeneracy stemming from group-theoretical symmetry argument is limited. The very viewpoint of the topological symmetry disclosed the possible occurrence of organic polymer superpara- and ferro-magnets, followed by conceptual advances particularly in spin chemistry. It is important to realize that the through-bond approach is based on the topological nature of the through-bond linkage modes of ***π***-conjugated spin systems.

Figure 2 shows the first documented band-structure calculation revealing the possible occurrence of ferromagnetic ground states for purely organic polymeric spin systems.2a),2c) The particular topology of ***π***-conjugated electron network in alternant hydrocarbon-based polymeric systems, i.e., *meta*- or 1-,3-,5-connectivity gives rise to the superdegeneracy4),6d) of half-filled NBMO’s in their ground state as depicted for an *m*-benzyl radicalbased one-dimensional magnetic polymer in Fig. 2. This symmetry requirement is interrelated to topologically controlled robust dynamic spin polarization of the systems (topological ***π***-spin polarization approach).6d) The argument for this can be rationalized by a DODS-based picture with one-center electron repulsion taken into account. The argument exactly corresponds to an MO description of molecular Hund’s rule (see the next section). It was a logical consequence that this approach predicted the occurrence of super high-spin macromolecules having extremely large spins or polymeric superpara- and ferro-magnetic polymers as shown in Fig. 1.

The use of topological symmetry in acquiring organic high-spin ground states for finite systems and ferromagnetic states for infinite systems gives a completely different answer to the degree of orbital degeneracy in organic molecular systems. In this context, ***π***-topology-based organic magnetism has not been any analogues to atomic based magnetism nor molecule-based magnetism via through-space approaches.15) The low symmetry intrinsic to organic molecules is the essential reason why high-spin multiplicities of electronic ground states exceeding triplet had never appeared from organic substances until the first organic high-spin quintet molecule was detected in 1967.1) The molecular design of the quintet molecule was exactly performed by the use of the topological symmetry argument. The concept has frequently been utilized as the first guiding principle for molecular designing of organic magnetic materials and experimentally tested.4)–7) Until recently, efforts from both experimental and theoretical sides have been made to control spin alignment in organic molecules and to understand underlying mechanisms, particularly electronic spin structures of open-shell systems in the ground and low-lying excited states.2)–6),9)–14) All issues of organic magnetism constitute current spin chemistry and the focus of contemporary topics in science.

Recent conceptual advances related to organic finite systems2)–6),23) have taken their crucial part in many aspects of the development of the research fields. Nevertheless, conceptual advances focusing on infinite or extended organic polymeric spin systems have not been made much so far.2a)–2e),13),14) The understanding inherent in the infinite spin systems serves the purposes of materials challenges and molecular designs for novel organic intriguing magnetic materials.4) In the issues of the infinite or extended open-shell systems, particular importance has been given to the exposition of the fundamental bases of polymeric organic magnetics (through-bond approach) such as molecular designs by utilizing the topological superdegeneracy of traveling wave orbitals (crystal orbitals), the dimensionality in spin structure, and the topology of inter-polymer contacts (inter-polymer connectedness) in terms of electronic band-structure calculations based on traveling wave approach (crystal orbital approach). These treatments have also described the first attempt to expound organic infinite polymeric open-shell systems in terms of VB approach; the VB picture has been obtained from the band-structure calculations, for the first time.4),6d)

### Intramolecular spin alignment in *π*-conjugated systems: The first principle of spin alignment in organic systems

Intramolecular spin alignment in homoatomic ***π***-conjugated systems such as hydrocarbons is governed by topologically controlled dynamic ***π***-spin polarization. The dynamic spin polarization underlies molecular Hund’s rule which is a molecular version of Hund’s rule. Empirical Hund’s rule cannot be allowed to apply to molecular systems without quantum chemistry justification. The simple extension of Hund’s rule is invalidated. Statements referring to “violation of Hund’s rule” appearing in the field of organic magnetism are misleading in this context. There is no violation of Hund’s rule.

**Fig. 4:**
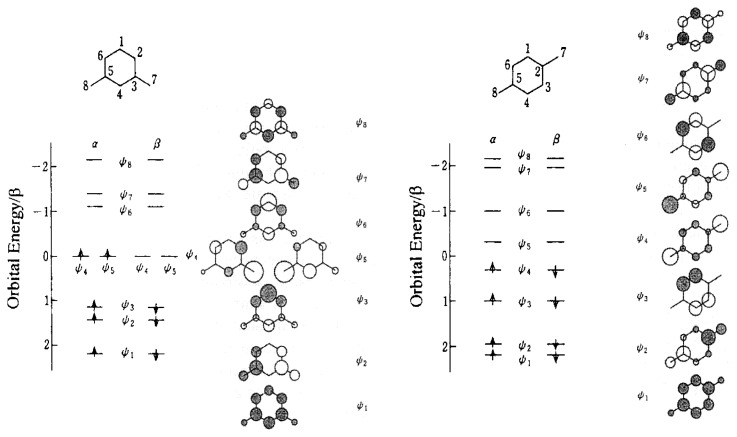
Intramolecular spin alignment by topologically controlled ***π***-spin polarization. Electronic structures of *m*-quinodimethane (left) and *p*-quinodimethane (right) in terms of DODS representation.

Figure 4 depicts simple molecular orbitals and electron configurations on the basis of different-orbitals-for-different-spins (DODS) representation for two ***π***-topological isomers of quinodimethane in their ground state. For the *m*-isomer two degenerate non-bonding ***π***-MO’s of zero energy in units of ***β*** (resonance integral) appear in the ***α***-orbital (and ***β*** -orbital). Appearance of these orbitals originates in the *meta* connectivity of ***π***-conjugation. In this context, this degeneracy is called “topological degeneracy” in contrast to ordinary orbital degeneracy due to geometrical symmetry. On the other hand, for the *p*-isomer the degeneracy is lifted and halves of NBMO’s rejoin bonding and antibonding ***π***-MO’s with a small HOMO-LUMO energy gap. For the *m*-isomer an ***α***-spin occupying ***Ψ***_4_*^α^* polarizes ***α***-spins of the bonding ***π***-MO’s at 2-, 4-, 6-, 7-, and 8-carbon sites. If an additional ***α***-spin occupies ***Ψ***_5_*^α^*, it causes additional ***α***-polarization for the bonding MO’s at the same carbon sites as depicted in the molecular orbital picture of ***Ψ***_5_. Thus, the polarization effect is additive (dynamic), leading to a ground-state triplet for the *m-*isomer. 24) If the additional spin occupies ***Ψ***_5_*^β^*, it causes ***β***-polarization at the same carbon sites, leading to competitive polarization in the ***π***-electron network. This destabilizes a spin-singlet state.

For the *p*-isomer a triplet excited state nearby a ground-state singlet state is expected to occur because of the small energy gap. Comparing an electron configuration |***Ψ***_1_*^α^****Ψ***_2_*^α^****Ψ***_3_*^α^****Ψ***_4_*^α^****Ψ***_1_*^β^****Ψ***_2_*^β^****Ψ***_3_*^β^****Ψ***_4_*^β^*|(*S* = 0) with |***Ψ***_1_*^α^****Ψ***_2_*^α^****Ψ***_3_*^α^****Ψ***_4_*^α^****Ψ***_5_*^α^****Ψ***_1_*^β^****Ψ***_2_*^β^****Ψ***_3_*^β^*|(*S* = 1), additive spin polarization stabilizes the latter configuration. At the same time the latter is destabilized in terms of kinetic energy since the ***α***-electron occupies ***Ψ***_5_*^α^* of higher energy. Eventually, the former configuration (*S* = 0) gives the ground state for the *p*-isomer, but with a thermally accessible triplet excited state because of the small HOMO-LUMO gap. The situation is the same for the corresponding *o*-isomer as that of the *p*-isomer. Dominant additive polarization favors high-spin ground states, while competitive one stabilizes low spin states. This high spin preference in terms of the argument of electron polarization at each carbon site is underlain by electron repulsion on one center.

The multiply additive polarizations originating in *meta*- or 1,3,5-connectivity of benzene rings are specifically termed “***π***-topological symmetry rule” for high spin preference by the author. The rule has been widely used as the first principle for molecular design of organic high-spin systems of ***π***-conjugation and theoretical interpretation of their electronic structure. As described above, “topologically controlled dynamic spin polarization” has given a rationale for the rule.

The above dynamic spin polarization approach is applicable to heteroatomic ***π***-conjugation systems, in which topological complete degeneracy is lifted. If robust dynamic ***π***-spin polarization is established in molecular design, a version of topology rule for heteroatomic ***π***-conjugation is validated.25),26) Otherwise, a simple extension or application of “topology rule for homoatomic ***π***-conjugation” is invalidated. Particularly, due care should be taken for spin prediction for systems with ionic heteroatoms in the conjugation.6d) Also, the dynamic spin polarization approach can be invoked to interpret intramolecular spin alignment in electronically excited states of ***π***-conjugated systems or ground states with pluri-charge, taking configuration interactions into account.24c)

### Band structures of one- and two-dimensional polymeric magnetic systems with topologically controlled *π*-spin polarization

#### 1. Traveling wave/crystal orbital approach

The first documented band-structure treatment2a),2c) was followed by theoretical studies of the possible occurrence of band structures from organic magnetic polymers or extended spin systems in various levels of approximation.2e),13),14),27) The first treatment, which proved the unlimitation of the degree of ***π***-topological orbital degeneracy for a one-dimensional ferromagnetic polymer, had appeared at earlier times before crystal orbital approach was established in polymer chemistry and quantum chemistry. We stress that in some aspects of electronic spin structures of organic polymeric systems simple and intuitive extensions obtained from a finite system to its infinite polymeric system do not work. We also stress that the theoretical model introduced in this section predicts essential general features of magnetic properties of the polymeric spin system which yield crucial aspects for its molecular designing, despite the fact that simplified theoretical models only represent coarse qualitative features of physical properties.

Due care and attention are required when the present method applies to non-alternant hydrocarbon-based polymeric spin systems in order to predict their potentiality as novel magnetic functionality materials. Introduction of hetero-atomic spin sites can be mostly treated as a trivial extension. The model introduced in our theoretical work is the simplest possible version for band-structure calculations of organic magnetic polymers and their inter-polymer interaction. The model is called traveling wave or crystal orbital approach. In the following, we skip a tedious description of the calculation model4), 26) and only illustrate the general features of the band structures we consider typical one-dimensional polymeric spin systems. Finally, to foresee underlying features of band structures due to inter-polymer interactions taking place in higher dimensional magnetic polymeric systems we advance the traveling wave approach to quasi two-dimensional magnetic polymeric systems.

We stress that the traveling wave approach applies to a simplified version, which is able to illustrate general features of infinite organic magnetic systems in contrast to finite ones. The approach here aims to derive band structures of the infinite polymeric spin systems governed by the topological nature of spin polarization, specifying microscopic descriptions of segments and interpolymer interactions (the topology of inter-polymer contacts).4)

#### 2. One-dimensional infinite or extended polymeric *π*-spin systems

**Fig. 5:**
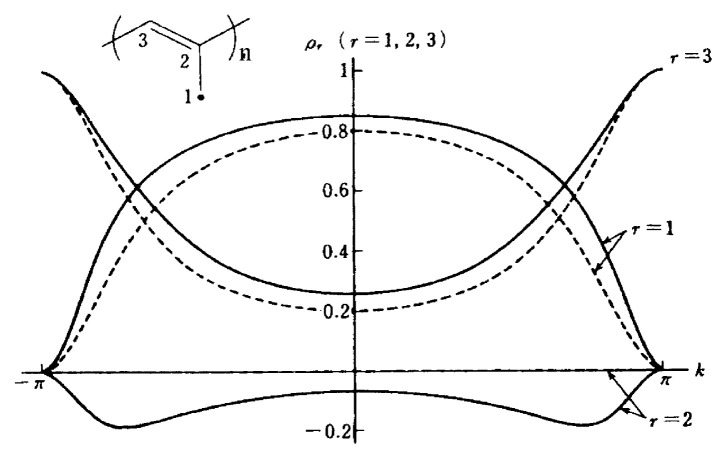
k-Space representation of the ***π***-spin density ***ρ***_r_ (r = 1,2,3) on the carbon site r of the 1D polymeric spin system (see Fig. 3). Solid and broken curves denote the calculated ***π***-spin densities with and without electron correlation, respectively.

The results show that the spin characteristic of a hydrocarbon-based polymeric model given in the inset of Fig. 5 cannot be simply attributed to the side-chain 1/2-spin (at site 1) type, but a spin characteristic of whole one 1/2-spin on the backbone chain type features in the infinite system. Figure 5 clearly illustrates the underlying nature of polymeric magnetic systems which cannot be predicted from intuitive considerations of finite magnetic molecules. The behavior of the spin density distribution of polymeric spin systems with extended ***π***-conjugation becomes much more complicated, featuring in the organic magnetic polymer systems. This complication arises from the topological nature of intra- and inter-polymer interactions in higher-dimensional structure and will be illustrated later.

Instead of the detailed description for the *m*- and *p-*benzyl-based polymers (see Fig. 1 (A) and Fig. 2), we briefly summarize the systems where the spin site 1 of the 1D polymer above is heteroatomic or comprises an inner segment structure with more or less spin polarization such as various pheoxyl radicals and phenyl nitroxides. Band-structure calculations in terms of the traveling wave approach predict as follows. If the systems are characterized by rather weak spin polarization and their spin site is hetero-atomic, their nonbonding band is expected to undergo stabilization below zero energy, but antiferromagnetic spin alignments in the k = ±***π*** region hamper the ferromagnetic property of the systems. In some cases with topologically controlled but weak spin polarization, the systems only show paramagnetism since inter-polymer antiferromagnetic contacts dominate intra-polymer ferromagnetic spin alignments. On the other hand, some interesting hetero-atomic effects feature in the relative order of bonding and nonbonding bands. The hetero-atomic effect appears in diminishing their band energy gaps between the NBCO and the near-by BCO, predicting modulations of thermal and optical properties of magnetic polymer systems. Systematic studies in this direction of the research on the experimental side will be required.4),28)

#### 3. Two-dimensional infinite or extended polymeric *π*-spin systems: topology of inter-polymer contacts (Inter-polymer connectedness)

**Fig. 6:**
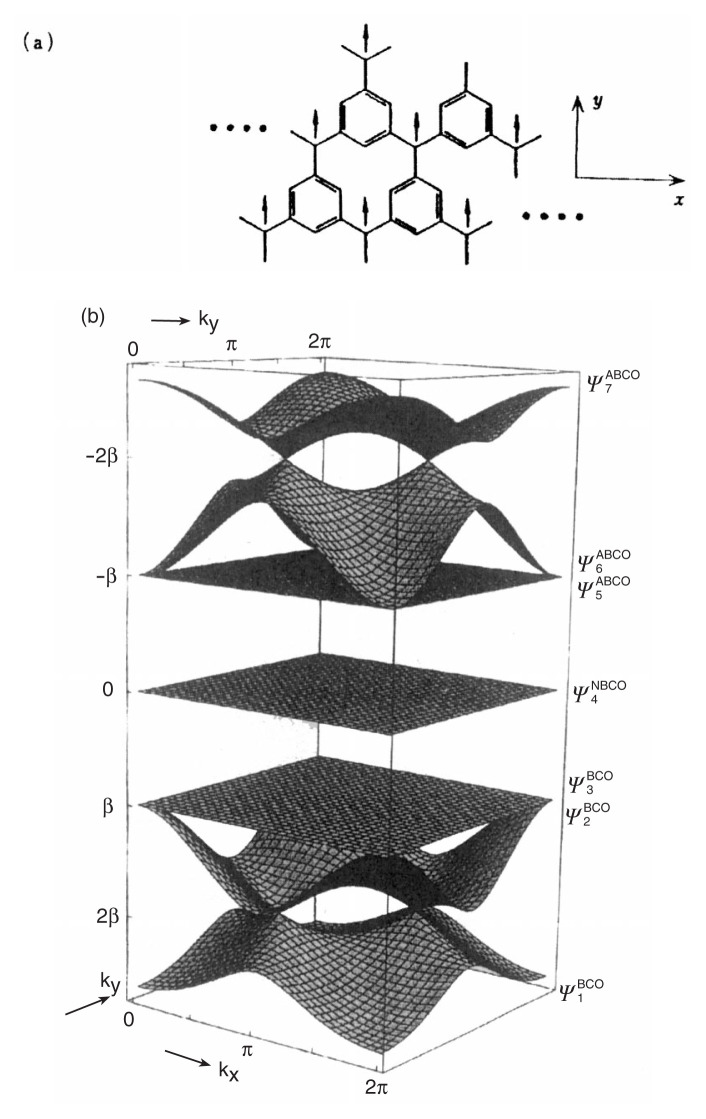
2D **k**-space representation of the band structure of the first two-dimensional polymeric model spin system for organic ferromagnets and superparamagnets. (a) The 2D polymer. (b) The NBCO appearing at zero energy manifests an infinitely thin bandwidth and delocalized nature (b). Infinitely thin bandwidths feature also in the lowest unoccupied ABCO and the highest occupied BCO.

Finally, we consider two-dimensional magnetic polymer systems in terms of the traveling wave approach, focusing on inter-polymer connectedness. Figure 6 illustrates in the **k**-space the band structure of the first two-dimensional ferromagnetic model polymer with topologically controlled robust spin polarization. The band structure manifests the *C*_3_ translation symmetry of the system. The NBCO appearing at zero energy with both an infinitely thin band-width and delocalized nature corresponds to the highest occupied band, which accommodates N parallel ***π***-spins in the ground state. Because of the robust spin polarization stemming from the topology of the ***π***-conjugation of the segment this two-dimensional polymer is expected to be a high-*T*c organic ferromagnet or a superparamagnet in certain materials conditions. Also due to the particular topology of the ***π***-conjugation and inter-segment linkage mode the lowest antibonding and highest bonding bands are characterized by infinitely thin bandwidths. Both the probability amplitudes on the carbon site in each crystal orbital and the spin density distribution in the zero energy traveling wave (NBCO) are modulated in a complex fashion in the two-dimensional **k**-space, closely depending on the inter-segment linkage mode in this model polymer.

In order to gain a physical insight of general features of this modulation, the most possibly simplified two-dimensional model polymer can be in quasi two-dimensional one which is comprised of the *m*-topological polymer in Fig. 2, and which has an antiferromagnetic contact between the two nearest-neighboring segments. In this quasi two-dimensional polymer, the N-fold superdegenerate nonbonding zero-energy band with N delocalizing ***π***-electrons still keeps an infinitely thin bandwidth, predicting the ferromagnetic ground state and possible purely organic superparamagnets in certain materials conditions. On the other hand, the lowest antibonding and highest bonding bands are strongly modulated through the antiferromagnetic inter-polymer (inter-1D chain) interaction and they are not superdegenerate any longer, resulting in finite band-widths for both of the bands.28)

## The first organic high-spin molecule in the spin-quintet ground state

**Fig. 7:**
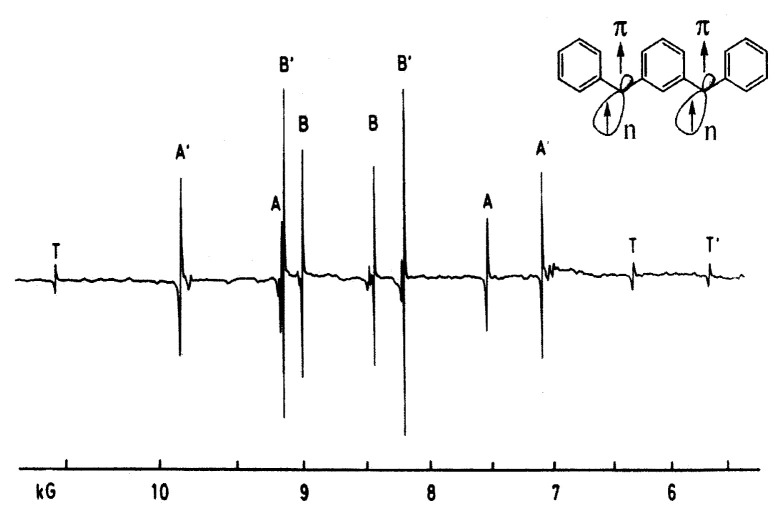
Electron spin resonance fine-structure spectrum observed at 77 K form *m*-phenylenebis(phenylmethylene) oriented in a benzophenone single crystal. The microwave frequency used is at K-band (23.942 GHz). The primed and unprimed capitals designate magnetically non-equivalent spin-quintet molecules with static magnetic field oriented in the crystallographic *ab* plane. T and T′ denote the signals from a triplet state of the byproduct (diphenylmethylene derivative).1a)

The first documented organic high-spin entity is *m*-phenylenebis(phenylmethylene) in the quintet ground state,1a) which was designed to stabilize by introducing extended ***π***-conjugation and two one-centered orthogonal singly occupied orbitals into *m*-quinodimethane (see Fig. 1). The molecular design was made by the first principle of spin alignment for organic systems. Figure 7 shows a pair of four ESR allowed transitions with the integrated intensity ratio of A(A′): B(B′)≒2 : 3 and the separation of A(or A′): that of B(or B′)≒3 : 1, showing that the observed ESR transitions arise from a spin-quintet state. Temperature dependence of the transition intensity down to 2 K confirmed that the quintet state is a ground state. Complete analysis of the angular variation of the observed resonance fields was carried out, enabling us to determine the fine structure constants, *D* = + 0.07131 cm^−1^, *E* = ± 0.01902 cm^−1^ with *g* = 2.003 and *S* = 2, unequivocally. The fine-structure constants predicted by Higuchi1c) were reasonably comparable with the experimental ones. The first semiempirical calculation of the **D** tensor for organic high-spin systems was made to determine a cis-trans molecular conformation for *m-*phenylenebis( phenylmethylene) in the benzophenone crystal lattice as well as in organic glassy solvents. The spin density distribution of the quintet molecule was determined by invoking single-crystal ^1^H-ENDOR (Electron-Nuclear-Double-Resonance) spectroscopy,29a) comparing with theoretical results obtained by semiempirical molecular orbital calculations, for the first time. Absolute sign determination of the fine-structure constant *D*-value was demonstrated in the ENDOR spectroscopy. Thus, methodology for high spin chemistry was established out of all recognitions in spin science.29) In addition, the first quintet-quintet optical absorption and emission spectroscopy was observed, identifying the electronic structure of *m*-phenylenebis(phenylmethylene) in the photoexcited quintet state.29b)

## One- and two-dimensionally extended high-spin hydrocarbons as studied by single-crystal electron magnetic resonance

The established methodology for high spin chemistry have been applied to one- and two-dimensionally extended high-spin hydrocarbons up to a ground-state tridecet molecule (*S* = 6) and its molecular structure in benzophenone crystals was fully determined by single-crystal ESR.

A series of 1D and 2D extended high-spin hydrocarbons in the ground state as studied by electron magnetic resonance spectroscopy are shown in the following.

**Figure:**
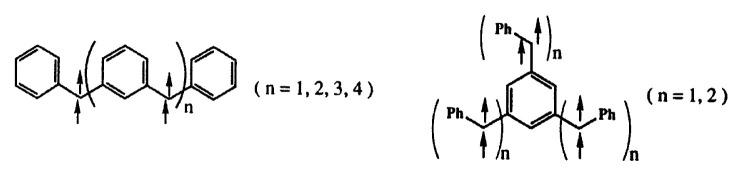


## VB approach and a generalized UHF Hubbard model applied to organic high-spin systems

**Fig. 9:**
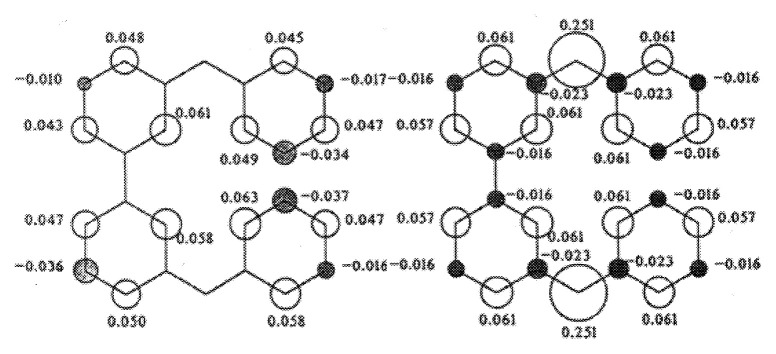
The comparison between the experimental spin density distribution (left) and theoretical ones (right) for (BP-3,3′-BPD) in the thermally accessible triplet state.

Quantum chemistry for organic high-spin systems has been hampered because of their intrinsic characteristics such as multi-centered molecular framework and multi-electron nature (electron correlation in open-shells). In order to identify electronic ground and nearby excited spin structures of the high spin systems, a large scale of configuration interactions are required, preventing MO approaches from the full understanding of the electronic nature of the high spin systems. We have applied, for the first time, a Heisenberg model Hamiltonian to organic high-spin molecules,11b),12) interpreting their electronic spin structures in the ground states as well as the nearby excited states by invoking the full CI method. Also, we have developed a generalized Hubbard model approach to sizable organic high-spin molecular systems and reproduced the experimentally determined spin density distribution of biphenyl-3,3′-bis(phenylmethylene) with the ground state singlet and thermally accessible a triplet state and a quintet state, demonstrating its usefulness as a theoretical tool in high spin chemistry.29c),29d) The comparison between the experimental spin density distribution and theoretical ones is depicted in Fig. 9. Biphenyl-3,3′-diylbis(phenylmethylene) (BP-3,3′-BPD) is the first organic high-spin entity that exemplifies the invalidity of the simple MO approach widely believed for spin alignment in organic open-shell systems in the ground state while the VB approach gives correct solutions.

## Intramolecular spin alignment in ionic systems of homoatomic *π*-conjugation

Among the diverse topics of molecular spin science underlying organic magnetics, charged organic high-spin systems have drawn particular attention not only because of their novelty but also because of their suitability as models for studying an interplay between spin polarization and charge fluctuation in organic molecules. This issue is crucial for designing magnetically intriguing new materials such as organomagnetic metals. Yamaguchi suggested from the theoretical side that oxidation of a high-spin ground state generates the possible hole-delocalization over an odd number of carbon sites, which drives a low-spin ground state via truncated spin polarization. In contrast to extensive studies of spin alignment in neutral molecule-based magnetics, only the above qualitative prediction was made for the spin alignment in monocationic high-spin systems.30) The spin prediction rule for neither mono-charged nor pluri-charged organic highspin systems has been established yet. An elaborate study of electronic and molecular structures of typical high-spin polycarbenes upon charging have been carried out,31) emphasizing that even intermediate ***π***-spin polarization dictates spin alignment in monoions of homoatomic high-spin hydrocarbons such as the polycarbenes.

**Fig. 10:**
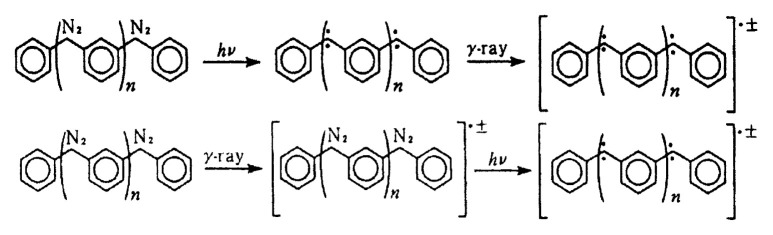
Generation of ionic high-spin entities from *m*-PBPM (*S* = 2) *via γ*-irradiation in frozen organic solutions. Two routes are shown.

Mono-charged high-spin states of polycarbenes have been generated by invoking *γ*-irradiation of frozen solutions according to the two routes as shown in Fig. 10. For the monoanion 2-MTHF was used as solvent and for the monocation *sec*-butyl chloride was used, respectively. 31)

### Spin states of monoionic m-phenylenebis (phenylmethylene), m-PBPM

**Fig. 11:**
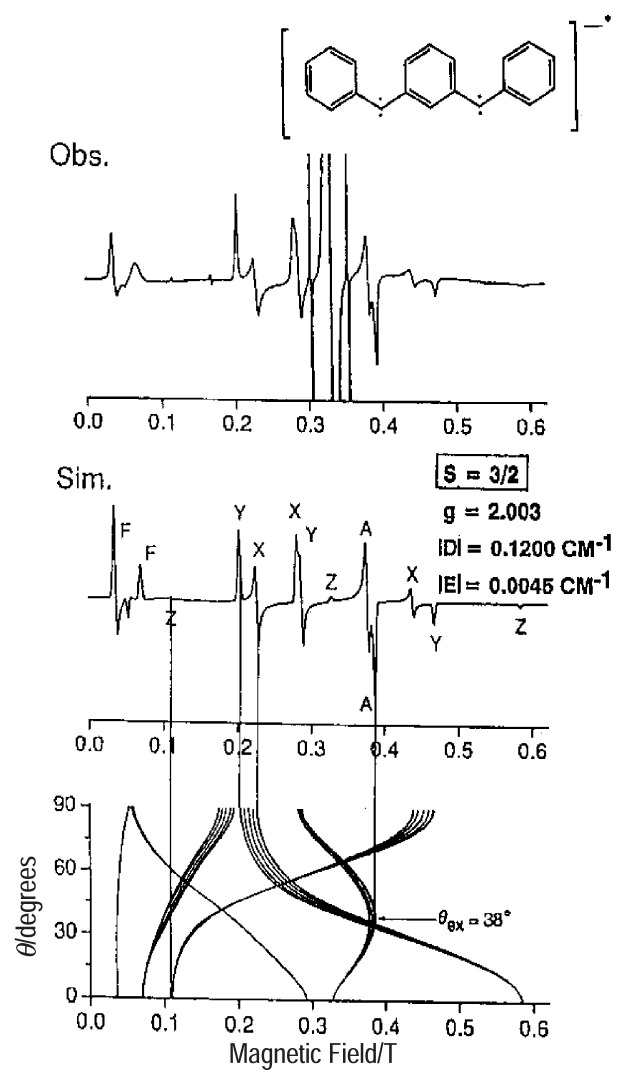
Observed and simulated ESR fine-structure spectra for the spin-quartet state of *m*-BPPM randomly oriented in a 2-MTHF glass at 77 K. Angular variation of the resonance fields is given for the complete spectral assignment (bottom).

The first detection and characterization of charged high-spin hydrocarbons in their ground state were carried out for *m*phenylenebis( phenylmethylene) (*m*-PBPM) (see Fig. 10), the first high-spin molecules whose spin alignment is dictated by robust ***π***-spin polarization.31) Figure 11 shows observed and simulated ESR fine-structure spectra from a monoanionic state (ground-state spin-quartet: *S* = 3/2) of *m*-PBPM randomly oriented in an organic rigid solvent. Angular dependence of resonance fields is also depicted in the figure for completing transition assignments.

From experimentally derived fine-structure constants and *g*-value it was concluded that an excess electron occupies ***π***-nonbonding MO’s and the monoion favors a *trans-trans* molecular conformation in contrast to a *trans-cis* form of the neutral parent quintet molecule in the ground state. The monocation of *m*-PBPM was also studied in detail by ESR and ENDOR spectroscopy, showing that an electron in the nonbonding ***π***- MO is removed upon ionization to yield the ***π***-cation.32) All the results show that the robust ***π***-spin polarization governs the spin alignment in the mono-charged *m*-PBPM to yield high spin preference in the ground state. A star-burst spin-septet hydrocarbon in the monoionic state was also studied, supporting the above conclusion.

### A prototypical high-spin hydrocarbon with a weak intramolecular exchange interaction, biphenyl-3,3′-diylbis(phenylmethylene), BP-3,3′-BPD

An attempt to ionize a prototypical high-spin hydrocarbon with a weak intramolecular exchange interaction, biphenyl-3,3*′*-diylbis(phenylmethylene) (BP-3,3*′*-BPD) with a singlet ground state was also carried out. Interestingly, BP-3,3*′*-BPD has a salient feature of the electronic spin structure with a nearby excited triplet and quintet state.2d),2f)

Upon charging, a doublet ground state with a thermally accessible quartet state was generated for both the monoanionic and monocationic BP-3,3*′*-BPD, showing that the intermediate ***π***-spin polarization predominates over the spin delocalization driven by the charge fluctuation. It turned out that the doublet-quartet energy gap is smaller in the cation than in the anion. This experimental result reflects the effect of the electron correlation as described below,33) indicating the explicit manifestation of electron correlation effects in the molecular framework with both charge and spin fluctuation.

**Fig. 12:**
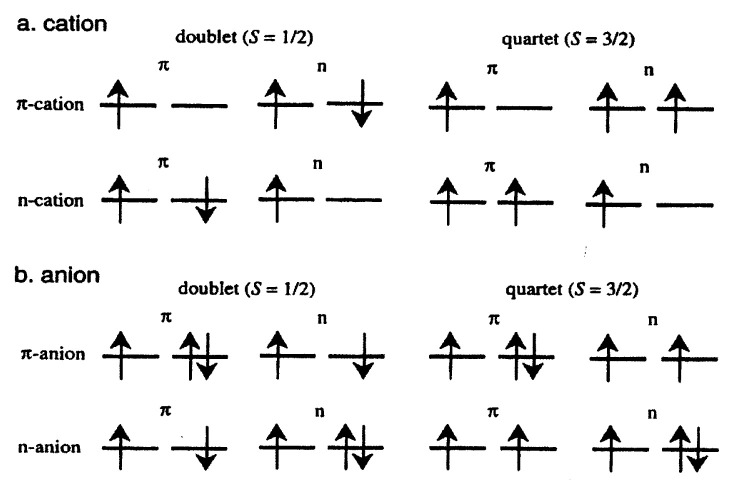
Electron configurations of the ***π***-cation and the n-cation (a) of BP-3,3′-BPM and the ***π***-anion and the n-anion (b), respectively.

The neutral BP-3,3*′*-BPM has four unpaired electrons in four nearly degenerate orbitals, two of which are ***π***-nonbonding orbitals and the other two are in-plane n-orbitals localized at the divalent carbon atoms. The removal of one electron from the ***π***-orbitals or from the n-orbital yields a ***π***-cation or an n-cation, respectively (see Fig. 12a). One of the four singly occupied molecular orbitals (SOMO’s) becomes a vacant orbital leading to a doublet or quartet state as far as these four SOMO’s are concerned. By comparing the electron configuration of the ***π***-cation and that of the n-cation, we can estimate that the energy difference between the ***π***- and the n-cation corresponds to that between the n- and the ***π***- orbital in either the doublet or quartet state; E*_π_*_-cation_ − E_n-cation_ = ***ɛ***_n_ − ***ɛ****_π_*. In general, the in-plane n-orbital has lower energy than the ***π***-orbital owing to the hybridization of the 2s orbital. Therefore the ***π***-cation is more probable than the n-cation.

In the case of the anion, the excess electron can occupy either the ***π***- or the n-orbital. The addition of one electron to the ***π***-orbital or the n-orbital generates a ***π***- anion or an n-anion, respectively (see Fig. 12b). In either doublet or quartet state, the energy difference between the ***π***- and the n-anion is given by E*_π_*_-anion_ − E_n-anion_ = (***ɛ***_n_ − ***π****_π_*) + (〈***ππ***|***ππ***〉 − 〈nn|nn〉). Whether the excess election of the anion is in the ***π***-orbital or in the n-orbital is dependent on the balance of the orbital energy of the ***π***- or the n-orbital and the electron repulsion between the ***π***- or the n-electrons. As a result, the preference of the ***π***-cation over the n-cation is concluded to be larger than that of the ***π***-anion over the n-anion. This difference of the number of electrons causes the effect of the electron correlation, which reflects the experimental result that the doublet-quartet energy gap Δ*E* is smaller in the cation than in the anion.

### Monoanion of the first prototypical intramolecular spin-frustrated system

The first prototypical example of charged through-bond spin-frustrated systems was studied34); the parent neutral molecule is 4,4′,4″-tris(diphenylmethylene)amine (N(*p*-DPM)_3_ (the ground-state triplet). This prototypical example was studied by ESR spectroscopy, concluding that the ground-state intermediate high-spin (*S* = 3/2) for the monoanion of N(*p*-DPM)_3_ was identified and an excess electron occupies the in-plane nonbonding MO predominantly localized at the divalent carbon site. For the corresponding monocation the highest spin state, *i.e.*, a sextet state (*S* = 5/2) is expected.

## Conclusions

The topological symmetry rule, as the first principle for molecular spin alignment in homo- and hetero-atomic finite systems of ***π***-conjugation is described in terms of the topological arguments underlain by dynamic ***π***-spin polarization. Organic molecular magnetism associated with the topological symmetry rule is treated as conceptual advance in natural science. The principle is extended to infinite molecular open-shell ***π***-systems, disclosing the general features of the electronic spin structures.

Methodology established for high spin chemistry is described, exemplifying the first organic high-spin molecule in the quintet ground state. Important theoretical advance in molecule-based magnetism is dealt with, showing the usefulness of a Heisenberg Hamiltonian approach and a generalized Habburd model approach as theoretical tools for the understanding of sizable openshell molecular systems.

Finally, various types of prototypical ionic high-spin states are dealt with to yield a basic understanding for an interplay between the ***π***-spin polarization and the spin delocalization in charged high-spin systems.

## Figures and Tables

**Fig. 3:**
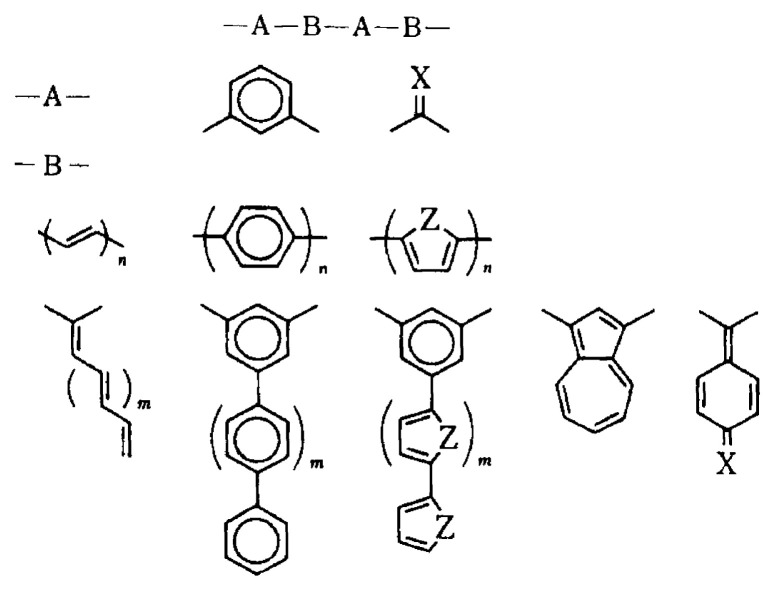
Model polymers for polaronic through-bond polymeric ferromagnets (Fukutome-Yamaguchi’s approach).

**Fig. 8:**
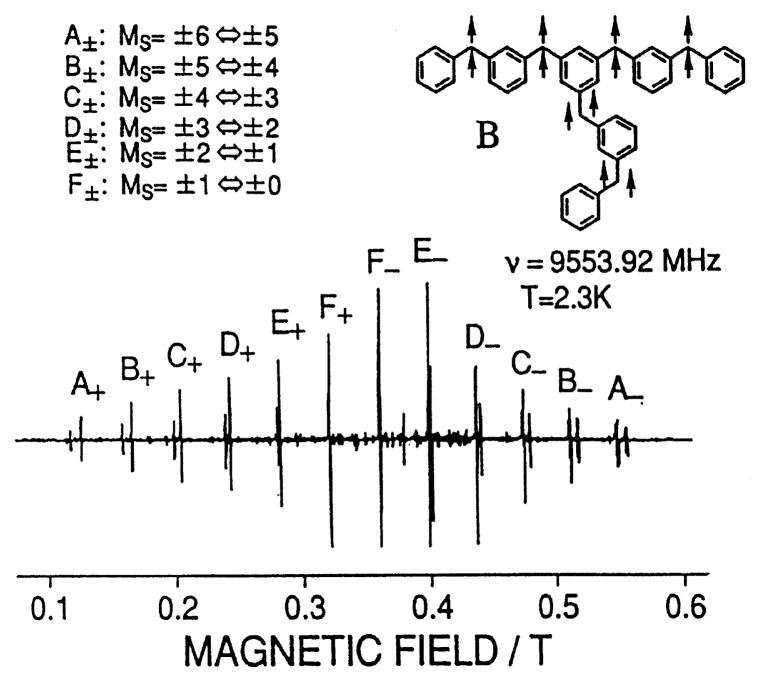
Fine-structure ESR spectrum (X-band) observed at 2.3 K from the ground-state tridecet molecule (see the inset) spectroscopy, as shown in Fig. 8. The tridecet molecule is markedly stable up to 230 K in the crystal lattice.

## References

[1] (a)Itoh K. (1967) Chem. Phys. Lett. 1, 235–238

[2] (a)Morimoto S. Tanaka F. Itoh K. Mataga N. (1968) Preprints of Symposium on Molecular Structure. Chem. Soc. Japan, Tokyo, pp. 76–77

[3] (a)McConnell H. M. (1967) Proc. T. A. Welch Found. Chem. Res. 11, 144–154

[4] Itoh K. Kinoshita M. (eds.) (2000) Molecular Magnetism: New Magnetic Materials. Gordon and Breach Publishers, Amsterdam (Kodansha, Tokyo as copublishers).

[5] (a)Chiang L. Y. Chaikin R. M. Cowan D. O. (eds.) (1989) Advanced Organic Solid State Materials, vol. 173, pp. 3–92

[6] (a)Miller J. S. Dougherty D. A. (eds.) (1989) Mol. Cryst. Liq. Cryst. 176, pp. 1–562

[7] (a)Takui T. Itoh K. (1990) Polyfile 27, 49–62

[8] (a)Miller J. S. Epstein A. J. (1991) Chemtech 21, 168–170

[9] Teki Y. Takui T. Itoh K. Iwamura H. Kobayashi K. (1986) J. Am. Chem. Soc. 108, 2147–2156, and references therein. 22175552 10.1021/ja00269a005

[10] (a)Borden W. T. Davidson E. R. (1977) J. Am. Chem. Soc. 99, 4587–4594

[11] (a)Klein D. J. Nelin C. J. Alexander S. Matsen F. M. (1982) J. Chem. Phys. 77, 3101–3108

[12] (a)Yamaguchi K. Toyoda Y. Fueno T. (1986) Chemistry 41, 585–595

[13] (a)Tyutyulkov N. Schuster P. Polansky O. E. (1983) Theor. Chim. Acta 63, 291–301

[14] (a)Nasu K. (1986) Phys. Rev. B 33, 330–338 10.1103/physrevb.33.330 9937914

[b15-pjab-80-029] 15Intermolecular spin alignment or through-space approach to organic magnetics is described in this review.

[16] (a)Kinoshita M. Turek P. Tamura M. Nozawa K. Shiomi D. Nakazawa Y. Ishikawa M. Takahashi M. Awaga K. Inabe T. Maruyama Y. (1991) Chem. Lett., 1225–1226 10.1103/PhysRevLett.67.746 10044978

[17] (a)Breslow R. (1982) Pure Appl. Chem. 54, 927–937

[18] Dormann E. Nowak M. J. Williams K. A. Angus R.O. Jr Wudl F. (1987) J. Am. Chem. Soc. 109, 2594–2599.

[19] Chiang L. Y. Johnston D. C. Goshorn D. P. Bloch A. N. (1989) J. Am. Chem. Soc. 111, 1925–1931.

[20] Torrance J. B. Bagus P. S. Johannsen I. Nazzal A. I. Parkin S. S. P. (1988) J. Appl. Phys. 63, 2967–2971.

[21] Fukutome H. Takahashi A. Ozaki M. (1986) Chem. Phys. Lett. 133, 34–38.

[22] (a)Kaisaki D. A. Chang W. Dougherty D. A. (1991) J. Am. Chem. Soc. 113, 2764–2766

[23] Longuet-Higgins H. C. (1953) J. Chem. Phys. 18, 265–274.

[24] (a)Kalafiloglou P. (1991) J. Chem. Educ. 68, 583–588;

[25] (a)Yoshizawa K. Takata A. Tanaka K. Yamabe T. (1992) Poly. J. 24, 857–866

[26] Fujinaga S. (1980) Molecular Orbital Theory. Iwanami Book Publisher, Tokyo.

[27] Hughbanks T. (1985) J. Am. Chem. Soc. 107, 6851–6857.

[28] Takui T., unpublished.

[29] (a)Takui T. Kita S. Ichikawa S. Teki Y. Kinoshita T. Itoh K. (1989) Mol. Cryst. Liq. Cryst. 176, 67–76

[30] (a)Yamaguchi K. Toyoda Y. Fueno T. (1987) Syn. Met. 19, 81–86

[31] (a)Matsushita M. Momose T. Shida T. Teki Y. Takui T. Kinoshita T. Itoh K. (1990) J. Am. Chem. Soc. 112, 4701–4702

[32] Matsushita M. Nakamura T. Momose T. Shida T. Teki Y. Takui T. Kinoshita T. Itoh K. (1993) Bull. Chem. Soc. Japan 66, 1333–1341.

[33] Nakamura T. Momose T. Shida T. Kinoshita T. Takui T. Teki Y. Itoh K. (1997) Mol. Cryst. Liq. Cryst. 306, 439–442.

[34] Nakazawa S. Sato K. Kinoshita T. Takui T. Itoh K. Nakamura T. Momose T. Shida T. Okuno T. Izuoka A. Sugawara T. (1997) Syn. Met. 85, 1753–1754.

